# Identification of *Streptococcus agalactiae* by fluorescent in situ hybridization compared to culturing and the determination of prevalence of *Streptococcus agalactiae* colonization among pregnant women in Bushehr, Iran

**DOI:** 10.1186/1471-2334-13-420

**Published:** 2013-09-08

**Authors:** Saeed Tajbakhsh, Marjan Norouzi Esfahani, Mohammad Emaneini, Niloofar Motamed, Elham Rahmani, Somayyeh Gharibi

**Affiliations:** 1 Department of Microbiology and Parasitology, Faculty of Medicine, Bushehr University of Medical Sciences, Moallem Street, P. O. Box 3631, Bushehr, Iran; 2 The Persian Gulf Tropical Medicine Research Center, Bushehr University of Medical Sciences, Bushehr, Iran; 3 Department of Microbiology, School of Medicine, Tehran University of Medical Sciences, Tehran, Iran; 4 Department of Community Medicine, Faculty of Medicine, Bushehr University of Medical Sciences, Bushehr, Iran; 5 Department of Obstetrics and Gynecology, Faculty of Medicine, Bushehr University of Medical Sciences, Bushehr, Iran

**Keywords:** *Streptococcus agalactiae*, Prevalence, Pregnant women, Fluorescent in situ hybridization, FISH

## Abstract

**Background:**

Pregnant women colonized by *Streptococcus agalactiae* (group B streptococci [GBS]) may transfer this microorganism to their newborns. *S. agalactiae* is an important cause of pneumonia, sepsis, and meningitis in newborns. Fluorescent in situ hybridization (FISH) is considered as a method of identification in the field of diagnostic microbiology. In this paper, we have designed a study to compare the DNA FISH after 7 h Lim broth enrichment and culturing for the identification of *S. agalactiae* and to determine the prevalence of vaginal colonization by *S. agalactiae* among pregnant women in Bushehr, Iran.

**Methods:**

Vaginal swab specimens were obtained from 285 pregnant women at 35 weeks or more than 35 weeks of gestation. The specimens were inoculated into Lim broth. In order to evaluate the sensitivity and specificity of GBS DNA FISH after 7 h Lim broth enrichment, the specimens were tested using both FISH and conventional culture methods. In addition, the prevalence of GBS colonization was determined.

**Results:**

Based on the results of this study, both the sensitivity and specificity of FISH were 100%. *S. agalactiae* was detected by both culture and FISH in 27 of the 285 pregnant women. Thus, the prevalence of GBS colonization was 9.5%.

**Conclusions:**

Since short-term (7 h) Lim broth enrichment followed by FISH using oligonucleotide probes showed a high sensitivity and specificity, this protocol is therefore a highly accurate and relatively rapid method for the detection of *S. agalactiae.* Our analysis suggests that the use of DNA FISH to screen for *S. agalactiae* colonization in pregnant women may be considered in the absence of GBS culture availability.

## Background

*Streptococcus agalactiae* (group B streptococci [GBS]) can be found in the genital and anorectal areas of many adults. This organism is one of the leading causes of invasive infections in newborns. Neonatal infections present as two distinct clinical pictures: early-onset disease, characterized by sepsis and pneumonia within the first week of life, and late-onset disease, with meningitis and sepsis between 7 days and 3 months of age. Maternal colonization by GBS is the most important risk factor for the development of invasive disease in their newborns [1]. Vaginal colonization by *S. agalactiae* occurs in approximately 5-40% of pregnant women [2], and GBS is transmitted to 40-70% of these colonized women’s newborns [3] during labor and birth [4]. Among colonized newborns, 1-3% develop disease [3,5]. Of these, 80% develop early onset disease [6]. The mortality rate ranges from 10% to 20%, and the infants who survive may suffer from mental retardation or visual loss [6,7].

The Centers for Disease Control and Prevention (CDC) recommend screening for GBS colonization in pregnant women at 35–37 weeks of gestation. Intrapartum antibiotic prophylaxis of the GBS-colonized pregnant women is recommended [8]. These recommendations significantly decrease the incidence of early-onset neonatal GBS disease [9]. An optimum preventive strategy results in the treatment of GBS-colonized pregnant women and minimizes the treatment of women who do not require it [10]. In order to optimize this strategy, a correct diagnosis of GBS colonization status for each individual pregnant woman is needed.

In Bushehr, pregnant women are not screened for antepartum GBS colonization. The isolation and identification of *S. agalactiae* via conventional culture methods is time-consuming, requiring 36–72 h. Moreover, culture-based identification requires multiple media and tests, as well as several technicians. Therefore, molecular methods, such as fluorescent in situ hybridization (FISH), could be helpful in the detection of GBS, especially at our institutions that have limited staff. The prenatal care program for pregnant women at our institutions includes at least eight evaluation steps at various periods of pregnancy; one of the evaluations is performed at 35–37 weeks of gestation; thus, FISH can be used for the identification of GBS in this stage of prenatal care.

FISH using oligonucleotide probes has been used for the rapid detection of GBS directly in vaginal swabs [7,11]. However, there is a discrepancy in the results. While Artz *et al*. reported FISH to be a highly sensitive technique (sensitivity of 98.3%) for the detection of *S. agalactiae* directly in specimens [7], Strus and colleagues could not confirm this sensitivity [11]. When we were designing our study, an article concerning the successful application of peptide nucleic acid (PNA) FISH for GBS detection after a short-term (7 h) Lim broth enrichment step was published; this procedure has been shown to be highly sensitive and specific [12]. However, in our laboratory, the FISH protocols have been established by using oligonucleotide (DNA) probes. Hence, a question was raised as to whether 7 h Lim broth enrichment followed by DNA-based FISH protocol results in a high sensitivity and specificity. Therefore, the present study aims to compare the DNA FISH after 7 h Lim broth enrichment and culture method for the identification of *S. agalactiae* and to determine the prevalence of vaginal colonization by *S. agalactiae* among pregnant women in Bushehr, south west of Iran.

## Methods

### Bacterial strains

The Deutsche Sammlung von Mikroorganismen und Zellkulturen (DSM) and the American Type Culture Collection (ATCC) reference strains, as well as other organisms used in this study were *S. agalactiae* (DSM 2134 and four clinical isolates), *S. pyogenes* (ATCC 19615), *S. sobrinus* (ATCC 27607), *S. sanguis* (ATCC 10556), a species of group D streptococci (clinical isolate), *Enterococcus faecalis* (ATCC 29212), *E. faecium* (clinical isolate), a beta-haemolytic strain of *Enterococcus* (clinical isolate), *Lactobacillus acidophilus* (ATCC 4356), and *Lactobacillus fermentum* (DSM 20052). These bacterial strains were used to check probe specificity. These strains were grown, harvested in exponential growth phase, and fixed with ethanol (Merck, Darmstadt, Germany), as described previously [13,14]. The fixed bacterial strains were then tested by FISH, as described below.

### Specimen collection

This study was approved by the Ethical Committee of Bushehr University of Medical Sciences. Between April 2010 and January 2011, vaginal swab specimens were collected from eligible pregnant women attending the two major hospitals in the city of Bushehr (south west of Iran), Bentolhoda and Salman Farsi. In our region, pregnant women did not permit us to obtain rectal swabs (because of religion, etc.); thus, we obtained vaginal specimens instead of recto-vaginal specimens. The samples were collected from vaginal introitus [10,15] using one sterile swab for each woman by one of four trained providers. The providers were blind to the delivery mode. The specimens were taken at 35–42 weeks of gestation. Women at a gestational age of 35 weeks or more who had not received antibiotics within two weeks prior to sampling were eligible. Of the eligible women, those who agreed to participate in this investigation and signed a consent form were included in the study. Eligible women were approached seven days per week during day and night shifts depending on the availability of providers to screen and consent women and to collect their samples. Women at a gestational age of less than 35 weeks, those who had received antibiotics within two weeks prior to sampling, and women who declined to participate were excluded from the study [2,16].

Each swab specimen was inoculated into 1 ml Lim selective enrichment broth (Todd Hewitt broth supplemented with colistin and nalidixic acid; Becton, Dickinson and Company, USA). In order to evaluate the sensitivity and specificity of GBS DNA FISH after 7 h Lim broth enrichment, the specimens were tested by using both FISH and conventional laboratory methods, such as gram stain, the detection of hemolysis type, catalase, bacitracin susceptibility and sulfamethoxazole – trimethoprim (SXT) susceptibility tests, hippurate hydrolysis, a CAMP (Christie-Atkins-Munch-Petersen) test, and a bile-esculin test.

### Short-term (7 h) Lim broth enrichment

One vaginal swab from each woman was enriched in Lim broth to use for both FISH and culturing. Short-term (7 h) Lim broth enrichment was performed as described by Peltroche-Llacsahuanga *et al*. [12], with the following minor changes: Briefly, the tips of the swab specimens were cut and then put into the microcentrifuge tubes (Greiner bio-one) containing 1 ml of Lim broth. The microcentrifuge tubes with submerged tips were shaken by slight vortexing them for 15 min and were then placed into a shaking incubator (Heidolph, Schwabach, Germany) for 6 h and 45 min at 37°C at 350 rpm. Afterwards, the swab tips were removed, and the microcentrifuge tubes were centrifuged at 8,000 rpm for 5 min. The sediment of each microcentrifuge tube was resuspended in 60 μl PBS. Of this, a volume of 50 μl was fixed with ethanol for testing via FISH. The remaining 10 μl was inoculated into a new Lim broth and incubated at 37°C for 18–24 h.

### Isolation of *S. agalactiae*

After 18–24 h, the Lim broth tubes were observed in order to check the turbidity; then, subculturing from the turbid broth cultures to blood agar plates was performed. According to the manufacturer’s directions, the Lim broth tubes that were not turbid were reincubated for an additional 24 h. Colonies on the blood agar that were suspected for *S. agalactiae* were subcultured for purity, and the final identification was carried out by conventional laboratory methods. Criteria such as a positive reaction with hippurate hydrolysis and a CAMP test and a negative reaction with the catalase and a bile-esculin test, as well as resistance to bacitracin (usually) and SXT, were considered for GBS.

### FISH

Oligonucleotide probes Saga and EUB338, which were synthesized and 5′-labeled (Metabion, Martinsried, Germany), were used in this study. The probe Saga (5′- GTA AAC ACC AAA CMT CAG CG -3′), which specifically targets and hybridizes to a 16S rRNA region of *S. agalactiae*, was used for the identification of this bacterium [14]. The 5′ end of Saga was labeled with fluorochrome Cy3, which emitted a red fluorescent signal. The probe EUB338 (5′- GCT GCC TCC CGT AGG AGT-3′), which hybridizes the 16S rRNA of almost all bacteria [17], was 5′-labeled with fluorochrome Fluo, which exhibits a green fluorescent signal.

FISH was performed according to a protocol described elsewhere [13,14]. Briefly, 20 μl of each enriched and fixed specimen or 10 μl of each fixed control bacterial strain were placed on glass slides and air dried. The dehydration step was carried out in an ascending ethanol series. For the permeabilization of bacterial cells, an enzymatic treatment was performed with 1 mg/ml lysozyme (Sigma, Steinheim, Germany) for 15 min. For hybridization, specimens or bacterial strains were covered with a hybridization buffer (0.9 M NaCl, 20 mM Tris–HCl [pH 8], 0.01% SDS, 20% formamide) containing both the probes EUB338-Fluo and Saga-Cy3. Slides were then incubated at 46°C for 90 min in the humid chambers. Afterwards, the slides were incubated in a washing buffer (20 mM Tris–HCl [pH 8], 0.01% SDS, 225 mM NaCl) at 48°C for 15 min. Subsequently, DNA was stained with 4′, 6-diamidine-2′-phenylindole dihydrochloride (DAPI; Roche, Mannheim, Germany). Finally, the slides were mounted with a fluorescent mounting medium (DAKO, Glostrup, Denmark) and observed with an epifluorescence microscope (Nikon 80i, Tokyo, Japan) equipped with a DS-5Mc-L1 digital camera. The time needed for the FISH technique itself was 3 h. The time-to-result was the 3 h for the FISH technique, plus the 7 h for the Lim broth enrichment, yielding a total time-to-result of 10 h.

For analysis of assay, the results of GBS DNA FISH after 7 h Lim broth enrichment were compared with results of conventional culture method.

## Results

Table 1 shows the results of the examination of the control strains via FISH using the oligonucleotide probes EUB338 and Saga. All bacterial strains were hybridized with EUB338. Only *S. agalactiae* strains were hybridized with Saga, which indicates the high specificity of this probe.

**Table 1:** Results of the examination of bacterial strains by FISH

**Bacterial strains**	**Source**	**Results of hybridization with probe**	
		**EUB338**	**Saga**
*S. agalactiae*	DSM 2134	+	+
*S. agalactiae*	Clinical isolate (from urine)	+	+
*S. agalactiae*	Clinical isolate (from vagina)	+	+
*S. agalactiae*	Clinical isolate (from wound)	+	+
*S. agalactiae*	Clinical isolate (from blood)	+	+
*S. pyogenes*	ATCC 19615	+	–
*S. sobrinus*	ATCC 27607	+	–
*S. sanguis*	ATCC 10556	+	–
A species of group D streptococci	Clinical isolate (from blood)	+	–
*E. faecalis*	ATCC 29212	+	–
*E. faecium*	Clinical isolate (from urine)	+	–
A β-haemolytic strain of *Enterococcus*	Clinical isolate (from vagina)	+	–
*L. acidophilus*	ATCC 4356	+	–
*L. fermentum*	DSM 20052	+	–

**Figure 1:**
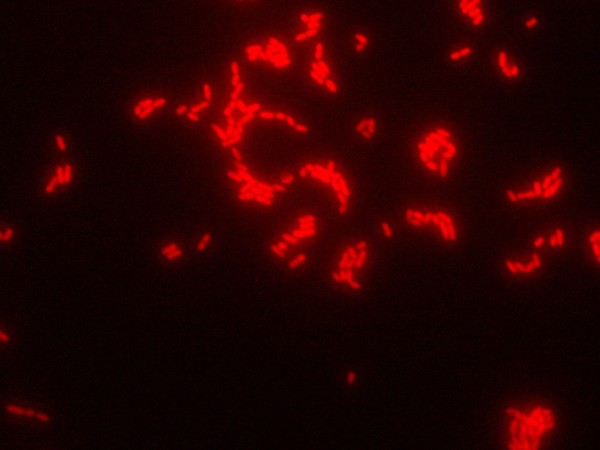
**Application of the DNA FISH method for the detection of *S. agalactiae.*** The red fluorescent signal of the GBS indicates hybridization with probe Saga-Cy3. Magnification, ×1000.

Two hundred and eighty-five vaginal specimens were enriched in Lim broth and examined by FISH and conventional culturing methods. In 27 of 285 specimens, *S. agalactiae* was detected via both FISH (Figure 1) and culturing methods. The remaining 258 specimens were negative for *S. agalactiae* according to both the FISH and culture results. Therefore, the sensitivity and specificity of GBS DNA FISH after 7 h Lim broth enrichment were both 100%. The prevalence of vaginal GBS colonization among pregnant women in Bushehr was 9.5%. The mean age of the pregnant women was 26.39 ± 5.33 years.

## Discussion

*S. agalactiae* remains an important cause of severe pneumonia, sepsis, and meningitis in neonates. GBS colonization of pregnant women increases the risk of the above-mentioned invasive diseases in neonates. To optimize the preventive strategy, a correct diagnosis of GBS colonization status for each pregnant woman is needed. The demand for the precise and rapid detection of infectious agents has made the application of molecular techniques, such as FISH, indispensable in diagnostic microbiology laboratories. In this study, we evaluated the sensitivity and specificity of GBS DNA FISH after 7 h Lim broth enrichment. In addition, the prevalence of GBS colonization was determined among pregnant women in Bushehr, south west of Iran.

In our study, the oligonucleotide probe Saga was used to identify *S. agalactiae*. In the studies performed by Trebesius *et al*. [14] and Kempf *et al*. [18], the Saga probe was examined with many target and non-target strains. These authors showed that the DNA probe Saga is highly specific for the detection of *S. agalactiae*. We examined the use of the Saga probe on some bacterial strains that may be present in the female genital tract [19,20]. Saga produced correct positive results with all target strains and correct negative results with all non-target strains. Therefore, Saga again proved to be a highly specific probe for the identification of *S. agalactiae.*

Some strains of *S. agalactiae* are non-hemolytic [19]. In many screening procedures, only beta-hemolytic colonies on blood agar plates are usually selected and further examined via conventional culture methods [7]. Therefore, non-hemolytic isolates will not be identified by these procedures. *S. agalactiae* DSM 2134, which is a non-hemolytic strain (as mentioned by the DSMZ), was hybridized with the DNA probe Saga. Thus, similar to GBS PNA FISH [12], non-hemolytic strains are also detectable by GBS DNA FISH.

*Lactobacillus* spp. are major members of the normal flora of the female genital tract. They are highly pleomorphic bacilli, which may appear in coccoid or spiral-shaped forms. The *L. acidophilus* complex constitutes the majority of the lactobacilli of the vagina, but other species, such as *L. fermentum*, have also been recovered [20]. To our knowledge, the probe Saga has not been previously tested on *Latobacillus* spp. We used *L. acidophilus* ATCC 4356 and *L. fermentum* DSM 20052 in our examinations, which showed the accurate function of Saga.

Specimens from 285 pregnant women were used to evaluate the sensitivity and specificity of DNA FISH for the detection of GBS from a 7 h Lim broth enrichment culture. No false-positive or false-negative results were observed. Therefore, both the sensitivity and specificity of our assay were 100%. Thus, DNA FISH, after short term (7 h) Lim broth enrichment, is a highly sensitive and specific procedure for the detection of *S. agalactiae.* This optimal sensitivity and specificity encourages us to perform the short-term Lim broth enrichment culture of swabs before FISH. Our results are in accordance with the results of the study done by Peltroche-Llacsahuanga *et al*. concerning the sensitivity and specificity of PNA FISH [12]. Also, in another investigation, conducted by Montague and colleagues, Lim broth enrichment followed by GBS PNA FISH showed high sensitivity and specificity (97.4% sensitivity and 98.3% specificity), but in their protocol, the incubation of the Lim broth was 24 h [21]. The preparation of PNA probes is expensive [22], and the inexpensive preparation of DNA probes is an important advantage of these probes over PNA probes.

Culture-based strategies require subculturing on blood agar plates, as well as multiple media and tests, for the definite identification of GBS. Sometimes, the application of these methods is not so easy because the growth of other microorganisms can cause some difficulty in the isolation of *S. agalactiae*, so staff must subculture several various suspect colonies for the sake of purity. In fact, they must perform identification tests for each suspect colony separately. However, the use of GBS FISH decreases these multiple handlings. Thus, it seems that FISH following short-term Lim broth enrichment is a useful detection protocol, particularly in settings with high volumes of specimens and limited personnel [12]. It should be emphasized that FISH is an easy and cost-effective technique that does not require specific equipment.

Nucleic acid amplification tests including polymerase chain reaction (PCR) assay for the detection of GBS that take 60–75 min, are also available. PCR is more rapid than our protocol; thus, PCR is a very helpful test for detecting intrapartum GBS carriers [23,24]. In fact, the rapidity of PCR is an advantage of this method over our protocol. On the other hand, the optimal sensitivity and specificity (both were 100%) of our procedure is an advantage of this technique over PCR. Therefore, our procedure is a more suitable test for antepartum screening for GBS carriage.

In our study, the prevalence of *S. agalactiae* colonization among pregnant women in Bushehr was found to be 9.5%. This is in accordance with some other studies in Iran that reported a prevalence of 9.1% in Kerman (southeast of Iran) [2] and Shiraz (south of Iran) [25]. However, a prevalence of 20.6% has been reported in Tehran [15], which is higher than our finding. Also, various results regarding the GBS prevalence in pregnant women have been reported in other countries, e.g., 10.6% in Turkey [26], 21% in The Netherlands [10], 23% in Dar es Salaam, Tanzania [16], and 25.3% in Ismailia, Egypt [27]. The differences in these prevalences could be due to differences in the sampling sites, diagnostic techniques, type of culture media, gestational ages at sampling, investigated populations, socio-economic statuses of study groups, geographical regions, ethnic or genetic factors, marital statuses, and sexual behaviors [10,15,16,26,27].

According to CDC guidelines, swabbing both the vagina and rectum increases the yield of GBS detection as compared with sampling the vagina without swabbing the rectum [8]. It has been shown that in some women *S. agalactiae* can be found only in rectal specimens [28]. In our study, the women did not permit us to obtain rectal swabs. Thus, we obtained the vaginal specimen alone. This might influence the prevalence of GBS colonization that was achieved in the present study. GBS colonization could have been found to be more prevalent among our study population if recto-vaginal swabs had been collected. Similar to our investigation, in some other studies, only genital specimens were examined [5,12,15,21,27].

## Conclusions

In conclusion, since 7 h Lim broth enrichment followed by FISH using oligonucleotide probes showed a high sensitivity and specificity, this procedure is a highly accurate and relatively rapid method for the detection of GBS. Our analysis suggests that using DNA FISH to screen for GBS colonization in pregnant women may be considered in the absence of GBS culture availability; larger studies are needed to further evaluate its clinical utility.

## Abbreviations

ATCC: American type culture collection; CAMP: Christie-Atkins-Munch-Petersen; CDC: Centers for disease control and prevention; DAPI: 4′, 6-diamidine-2′-phenylindole dihydrochloride; DSM: Deutsche Sammlung von Mikroorganismen und Zellkulturen; FISH: Fluorescent in situ hybridization; GBS: Group B streptococci.

## Competing interests

The authors declare that they have no competing interests.

## Authors’ contributions

ST conceived of the study and designed it, carried out the laboratory tests, participated in the acquisition of data, participated in the analysis and interpretation of data, and drafted the manuscript. MNE carried out the laboratory tests, participated in the acquisition of data, and participated in the analysis and interpretation of data. ME participated in the analysis and interpretation of data. NM participated in the analysis and interpretation of data, and helped to draft the manuscript. ER participated in the data acquisition and helped to interpret the data. SG participated in the laboratory tests and the acquisition of data. All authors read and approved the final manuscript.

## Pre-publication history

The pre-publication history for this paper can be accessed here:

http://www.biomedcentral.com/1471-2334/13/420/prepub
